# Changing frequency of fluctuating light reveals the molecular mechanism for P700 oxidation in plant leaves

**DOI:** 10.1002/pld3.73

**Published:** 2018-07-23

**Authors:** Ginga Shimakawa, Chikahiro Miyake

**Affiliations:** ^1^ Department of Biological and Environmental Science Faculty of Agriculture Graduate School of Agricultural Science Kobe University Kobe Japan; ^2^ Core Research for Environmental Science and Technology Japan Science and Technology Agency Tokyo Japan

**Keywords:** fluctuating light, P700 oxidation, photosynthesis, reactive oxygen species

## Abstract

Natural sunlight exceeds the demand of photosynthesis such that it can cause plants to produce reactive oxygen species (ROS), which subsequently cause photo‐oxidative damage. Because photosystem I (PSI) is a major source of ROS, plants actively maintain the reaction center chlorophyll of PSI(P700) oxidized under excessive light conditions to alleviate the ROS production. P700 oxidation is universally recognized in photosynthetic organisms as a physiological response to excessive light. However, it is still poorly understood how P700 oxidation is induced in response to fluctuating light with a variety of frequencies. Here, we investigated the relationships of photosynthetic parameters with P700 oxidation in *Arabidopsis thaliana* under a sine fluctuating light with different frequencies. As the photon flux density of the light increased, P700 was oxidized concurrently with the chlorophyll fluorescence parameter qL unless the electron acceptor side of PSI was limited. Conversely, we did not observe a proportional relationship of non‐photochemical quenching with P700 oxidation. The mutant *crr‐2*, which lacks chloroplast NADPH dehydrogenase, was impaired in P700 oxidation during light fluctuation at high, but not low frequency, unlike the *pgrl1* mutant deficient in PGR5 and PGRL1 proteins, which could not oxidize P700 during light fluctuation at both high and low frequencies. Taken together, our findings suggested that the changing frequency of fluctuating light reveals the tracking performance of molecular mechanisms underlying P700 oxidation.

## 1 INTRODUCTION

Plants are vulnerable to photo‐oxidative damage by reactive oxygen species (ROS) that originate from the photosynthetic electron transport system. During the light reaction of photosynthesis, photon energy is absorbed by the chlorophyllin photosystem (PS) I and II on the thylakoid membrane, thereby driving photosynthetic linear electron transport from PSII to PSI through the plastoquinone (PQ) pool, the cytochrome *b*_6_/*f* complex (Cyt *b*_6_/*f*), and plastocyanin. On the electron acceptor side of PSI, NADP^+^ is reduced to NADPH with electrons from PSI via ferredoxin (Fd) and Fd‐NADP^+^ reductase. Conversely, in PSII, H_2_O is oxidized on the luminal side of the thylakoid membrane; in Cyt *b*_6_/*f*, the Q‐cycle pumps stromal H^+^ to the luminal side, both of which establish a proton gradient (ΔpH) across the thylakoid membrane to produce ATP via chloroplast ATP synthase. The resulting NADPH and ATP are utilized for CO_2_ assimilation in the Calvin–Benson cycle. Limitation during photosynthetic CO_2_ assimilation under low CO_2_ and high light intensity can cause accumulation of excess electrons in the photosynthetic electron transport system. The recent study simulated such conditions by applying repetitive short‐pulse illumination and found that PSI was rapidly inactivated (i.e. PSI photoinhibition) via ROS, including superoxide anion radical ($O_{2}^{-}$), hydroxyl radical (·OH), and singlet oxygen (${}^{1}O_{2}$; Sejima et al., 2014; Zivcak, Brestic, Kunderlikova, Sytar, & Allakhverdiev, 2015; Zivcak, Brestic, Kunderlikova, Olsovska, & Allakhverdiev, 2015; Takagi, Takumi, Hashiguchi, Sejima, & Miyake, 2016).

Usually, PSI photoinhibition is rarely observed, and plants can tolerate sunlight (2,000–3,000 μmol photons m^−2^ s^−1^) that far exceeds the demands of photosynthesis. Recently, Sejima et al. (2014) confirmed that ROS production is suppressed when the reaction center chlorophyll of PSI (P700) is maintained in its oxidized form (P700^+^), because of the decrease of P700 in the ground state, which is the source of both excess electrons and energy to produce ROS. The oxidation of P700 is recognized as a physiological response to excessive light conditions, such as highlight and low CO_2_ (Golding & Johnson, 2003; Klughammer & Schreiber, 1994; Miyake, Miyata, Shinzaki, & Tomizawa, 2005; Shimakawa, Ishizaki et al., 2017). The strategies to oxidize P700 have been utilized and diversified since the origin of oxygenic photosynthesis (Shaku, Shimakawa, Hashiguchi, & Miyake, 2016; Shimakawa, Shaku, & Miyake, 2016). These facts indicate that P700 oxidation is the strategy that prevents photo‐oxidative damage caused by ROS in PSI. In nature, plants should be suddenly exposed to an excess light because sunlight is fluctuating with different frequencies (e.g., the range from 0.2 to 50 s, and from 0.004 to 1 Hz; Pfitsch & Pearcy, 1989). Overall, plants develop various molecular mechanisms for P700 oxidation (i.e. the P700 oxidation system) to allow them to tolerate the strongly fluctuating sunlight.

The oxidation of P700 is controlled by diverse molecular mechanisms on both the electron acceptor and donor sides of PSI. On the acceptor side, photosynthetic CO_2_ assimilation and $O_{2}^{-}$ dependent alternative electron transport function as electron sinks, thereby contributing to P700 oxidation. In C_3_ plants, the flux of electrons to O_2_ is mainly driven by photorespiration (Hanawa et al., 2017; Sejima et al., 2016; Wiese, Shi, & Heber, 1998). Even although photorespiration is the largest alternative electron sink in land plants (except for C_4_ plants; Hanawa et al., 2017), it is not used as the main alternative electron sink by many prokaryotic and eukaryotic algae (Hayashi et al., 2014; Shimakawa et al., 2015; Shimakawa, Akimoto et al., 2016; Shimakawa, Matsuda et al., 2017). Cyanobacteria, which are the ancestors of chloroplasts in land plants, use flavodiiron proteins (FLV) to mediate electron flow to O_2_, instead of photorespiration (Helman, Barkan, Eisenstadt, Luz, & Kaplan, 2005; Helman et al., 2003; Shimakawa et al., 2015), and to alleviate photo‐oxidative damage (Allahverdiyeva et al., 2013; Shimakawa, Shaku et al., 2016; Zhang, Allahverdiyeva, Eisenhut, & Aro, 2009). The physiological functions of FLV are also conserved in basal land plants such as *Marchantia polymorpha* and *Physcomitrella patens* (Gerotto et al., 2016; Shimakawa, Ishizaki et al., 2017), probably owing to evolutionary and ecological circumstances (Shimakawa, Ishizaki et al., 2017). On the donor side of PSI, the suppression of electron transport into PSI causes P700 oxidation. Basically, photosynthetic linear electron flow is limited in the oxidation of reduced PQ (i.e. plastoquinol, PQH_2_) in Cyt *b*_6_/*f* (Anderson, 1992; Kirchhoff, Horstmann, & Weis, 2000; Schöttler, Tóth, Boulouis, & Kahlau, 2015; Stiehl & Witt, 1969; Yamori et al., 2011). However, in response to short‐term environmental fluctuations, the electron transport in Cyt *b*_6_/*f* is modulated by a regulatory mechanism, which is believed to be strongly associated with ΔpH in plant leaves. The acidification on the luminal side of the thylakoid membrane limits the electron transport in Cyt*b*_6_/*f* (Nishio & Whitmarsh, 1993) and induces the dissipation of excess photon energy as heat at PSII (i.e. qE quenching) reflected as non‐photochemical quenching (NPQ) of chlorophyll fluorescence (Ruban, 2016). The formation of ΔpH is coupled with total electron transport activity (Kanazawa & Kramer, 2002) and is adjusted by the proton conductance (gH^+^) of the chloroplast ATP synthase (Rott et al., 2011; Takizawa, Kanazawa, & Kramer, 2008) or other ion transporters (Armbruster et al., 2014). Furthermore, ΔpH formation is possibly promoted by cyclic electron transport (CET) around PSI (Allen, 2003; Nandha, Finazzi, Joliot, Hald, & Johnson, 2007). Conversely, the suppression of electron transport into PSI for P700 oxidation(i.e. reduction‐induced suppression of electron transport, RISE) has been observed in the cyanobacterium *Synechococcus elongatus* PCC 7942 (Shaku et al., 2016; Shimakawa, Shaku, & Miyake, 2018). In RISE, the suppression of electron transport in Cyt *b*_6_/*f* is triggered by the reduction of the PQ pool, but not the accumulation of ΔpH,which implies that RISE is caused by the suppression of the Q‐cycle in Cyt*b*_6_/*f* because of a shortage of oxidized PQ (Shaku et al., 2016). Furthermore, $O_{2}^{-}$ dependent alternative electron transport mediated by terminal oxidases on the thylakoid membrane can contribute to P700 oxidation on the electron donor side of PSI (Feilke et al., 2016; Shimakawa & Miyake, 2018). For the last two decades, P700 oxidation has been observed as a universal physiological response to excess light conditions and reported to be related to a variety of molecular mechanisms. Nevertheless, the molecular mechanisms underlying P700 oxidation in plant leaves are still poorly understood under fluctuating light with different frequencies.

This study aimed to assess the impact of changing frequency of fluctuating light on P700 oxidation and other photosynthetic parameters in the model C_3_ plant *Arabidopsis thaliana*. We used a physiological measuring system, in which the photon flux density (PFD) of actinic light (AL) oscillates as a sine‐like curve with different frequencies. We measured various photosynthetic parameters in this system and found that P700 oxidation basically exhibited a linear proportional relationship with the chlorophyll fluorescence parameter qL regardless of the frequency of the sine fluctuating light in wild‐type *A. thaliana* (Col‐0). To investigate the effect of the electron acceptor side limitation in PSI on the relationship, we analyzed the *A. thaliana* mutants, *pgrl1* and *crr‐2*. The mutant *pgrl1* is deficient in proton gradient regulation 5 (PGR5)‐like 1 protein (PGRL1), the membrane‐associated protein, and is impaired in the expression of the soluble protein PGR5 (DalCorso et al., 2008). Previous and recent studies have shown that the lack of PGR5 and PGRL1 causes electron acceptor side limitation in PSI and the inability to oxidize P700, resulting in PSI photoinhibition under excessive light conditions in C_3_ plants (DalCorso et al., 2008; Kono & Terashima, 2016; Munekage et al., 2002; Suorsa et al., 2012; Yamori, Makino, & Shikanai, 2016). Conversely, the mutant *crr‐2* is impaired in the expression of the *ndhB* gene and lacks chloroplast NADPH dehydrogenase (NDH; Hashimoto, Endo, Peltier, Tasaka, & Shikanai, 2003). The lack of NDH is known to have little effect on photosynthetic electron transport at steady‐state photosynthesis in C_3_ plants (Sazanov, Burrows, & Nixon, 1998). However, recent studies suggested that the induction of P700 oxidation is impaired in *crr* mutants of *A. thaliana* and *Oryza sativa*, owing to the severe limitation of electron transport on the acceptor side of PSI, under a rectangular fluctuating light (Kono & Terashima, 2016; Yamori et al., 2016). Based on these findings of these studies, we used *pgrl1* and *crr‐2* and subjected them to acceptor side limitation in PSI in *A. thaliana*. In this study, *crr‐2* could oxidize P700 following light fluctuation only when the frequency of the fluctuating light was low, indicating that this mutant lacks the ability for the rapid initiation of P700 oxidation under fluctuating light in *A. thaliana*.

## 2 METHODS

### 2.1 *Arabidopsis thaliana* growth conditions

Plants of *A. thaliana* wild‐type (Col‐0) and the mutants *pgrl1* and *crr‐2* were grown under long‐day conditions (16 hr‐light, 23°C, 100 μmol photons m^−2^ s^−1^, white fluorescent lamp/8 hr‐dark, 21°C)with a relative humidity of 60 ± 10%. Seeds were planted in pots that contained a 1:1 mix of vermiculite and Metro‐Mix 350 (Sun Gro Horticulture, Agawam, MA, USA), and 1000‐fold diluted Hyponex solution (Hyponex, Osaka, Japan) was used as a watering solution. Photosynthetic parameters were measured in the rosette leaves of the plants 4 weeks after germination.

### 2.2 Measurements of gas exchange, P700, and chlorophyll fluorescence

Gas exchange, P700^+^ absorbance, and chlorophyll fluorescence were simultaneously measured using a Dual/KLAS‐NIR spectrophotometer, a GFS‐3000, and a 3010 DUAL leaf cuvette (Walz, Effeltrich, Germany), following previously reported methods (Klughammer & Schreiber, 2016), in which ambient air was saturated with water vapor at 18.0 ± 0.1°C, and the leaf temperature was maintained at 25 ± 2°C. Red AL (630 nm) was supplied using a chip‐on‐board LED array. Long‐term (3–5 hr) dark‐adapted plants were used for the measurements.

Exchange of CO_2_ and H_2_O was measured using GFS‐3000 equipped with a 3010‐DUAL gas exchange chamber (Walz, Effeltrich, Germany). Atmospheric gas (40 Pa CO_2_/21 kPa O_2_) was used in the present study.

The redox state of P700 was estimated from four original pulse‐modulated dual‐wavelength difference signals in the range of near‐infrared (785–840, 810–870, 870–970, and 795–970 nm; Klughammer & Schreiber, 2016). The photosynthetic parameters of PSI were calculated from the redox state of P700 as follows (Inoue, Ogawa, & Shibata, 1973; Klughammer & Schreiber, 1994; Schreiber & Klughammer, 2008): quantum yield of photochemical energy conversion in PSI, $Y\left(I\right)=\left(P_{m}^{\prime }-P\right)/P_{m}$; quantum yield of non‐photochemical energy dissipation due to donor side limitation, Y(ND) = *P/P*_m_; quantum yield of non‐photochemical energy dissipation due to acceptor side limitation, $Y\left(\text{NA}\right)=\left(P_{m}-P_{m}^{\prime }\right)/P_{m}$; *P*m, total amount of photo‐oxidizable P700; $P_{m}^{\prime }$, maximum amount of photo‐oxidized P700 by a saturation pulse; and *P*, amount of photo‐oxidized P700 at steady‐state. The sum of the three factors [Y(I) + Y(NA) + Y(ND)] = 1. For the determination of $P_{m}^{\prime }$, a short‐saturation pulse (8,000 μmol photons m^−2^ s^−1^, 300 ms) was applied to the plant leaves and, for the determination of *P*_m_, the short‐saturation pulse was applied after 10 s illumination with a far red light (740 nm). In this study, we determined *P*_m_ before the measurements in Umibozu. Only for the data shown in Figure 4, *P*_m_ was determined again at 30 min (in the dark) after 1 hr Umibozu for comparison with the *P*_m_ value before the treatment in Umibozu. During Umibozu, AL was temporarily turned off for 1 s just after the application of the short‐saturation pulse for the determination of *P*_o_.

The photosynthetic parameters of PSII were calculated from chlorophyll fluorescence (>700 nm; Krause & Weis, 1984) as follows (Baker, 2008): PSII operating efficiency (quantum yield of photochemical energy conversion in PSII), Y(II) or $\phi_{\mathrm{PSII}}=\left(F_{m}^{\prime }-F^{\prime }\right)/F_{m}^{\prime }$; non‐photochemical quenching, $\text{NPQ}=\left(F_{m}-F_{m}^{\prime }\right)/F_{m}^{\prime }$; fraction of “open” PSII centers (with Q_A_ oxidized) on the basis of the lake model for the PSII photosynthetic apparatus, $\text{qL}=\left(F_{m}^{\prime }-F^{\prime }\right)/\left(F_{m}^{\prime }-F_{o}\right)$; *F*_o_, minimum fluorescence from a dark‐adapted leaf; $F_{o}^{\prime }$, minimum fluorescence from a light‐adapted leaf; *F*_m_, maximum fluorescence from a dark‐adapted leaf; $F_{m}^{\prime }$, maximum fluorescence from a light‐adapted leaf; and *F* ^′^, fluorescence emission from a light‐adapted leaf. Pulse‐amplitude modulated green measuring light (540 nm, >0.1 μmol photons m^−2^ s^−1^) was used to determine *F*_o_. To obtained *F*_m_ and $F_{m}^{\prime }$, we applied a short‐saturation pulse (8,000 μmol photons m^−2^ s^−1^, 300 ms). During Umibozu, AL was temporarily turned off for 1 s just after the application of the short‐saturation pulse for the determination of $F_{o}^{\prime }$. In this study, we determined both *F*_o_ and *F*_m_ in the dark‐adapted leaves before the measurements in Umibozu. Only for the data shown in Supporting Information Figure S2, *F*_o_ and *F*_m_ were determined again at 30 min (in the dark) after 1 hr Umibozu for the comparison of *F*_v_/*F*_m_ with the plant leaves before the treatment in Umibozu.

### 2.3 Statistical analysis

We used Student's *t*‐tests to detect differences. All statistical analyses were performed using Microsoft Excel 2010 (Microsoft, Washington, USA), Origin 2017 (Lightstone, Tokyo, Japan), and JMP8 (SAS Institute Inc., Tokyo, Japan).

## 3 RESULTS

### 3.1 Responses of photosynthetic parameters to light fluctuation in *slow* Umibozu

**Figure 1:**
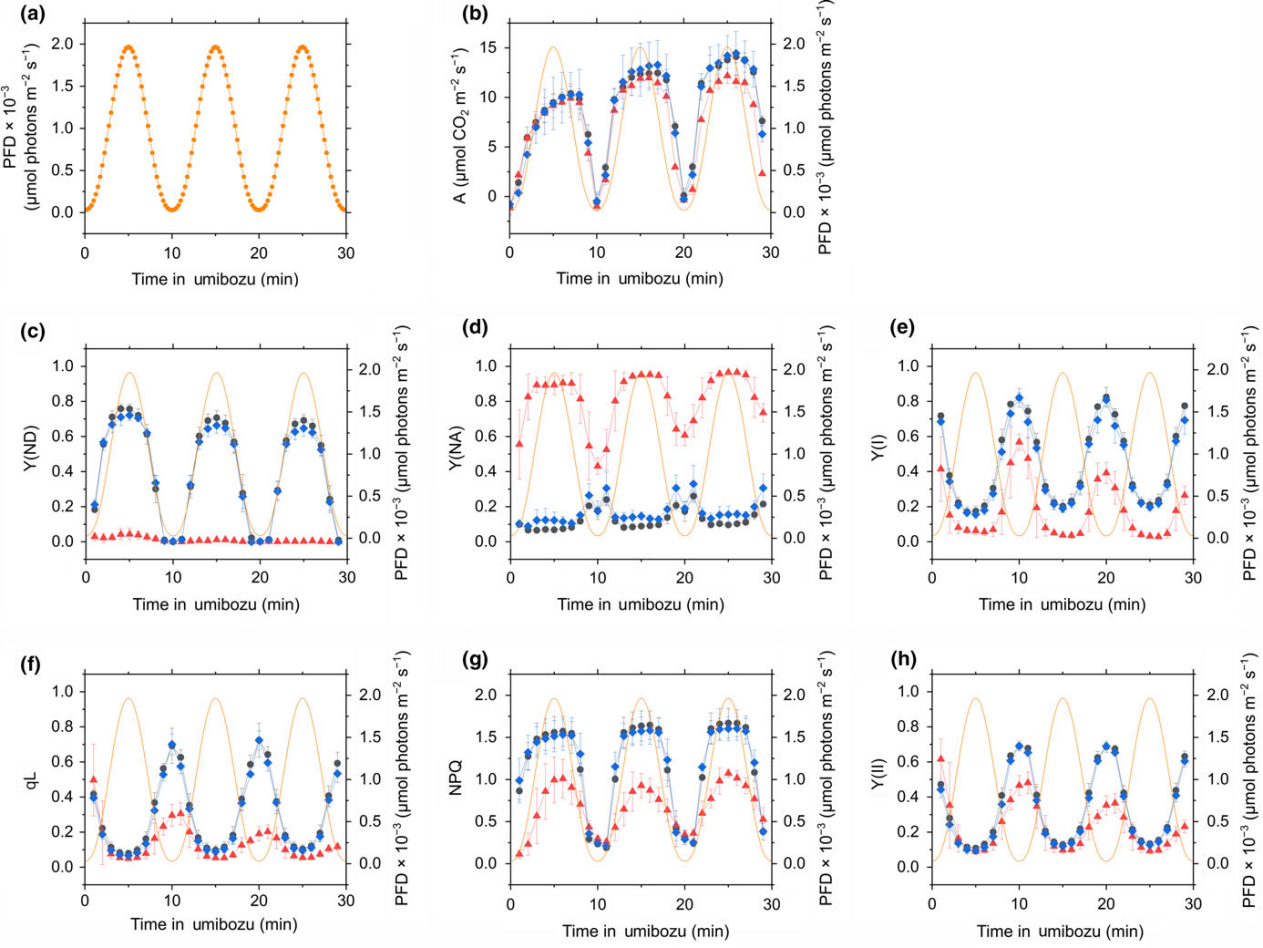
Time courses of photon flux density (PFD; a), net CO_2_ assimilation rate, A (b), Y(ND) (c), Y(NA) (d), Y(I) (e), qL (f), NPQ (g), Y(II) (h) during *slow* Umibozu in *Arabidopsis thaliana* wild‐type (Col‐0, black circles) and the mutants, *pgrl1* (red triangles) and *crr‐2* (blue diamonds). Orange lines indicate PFD. Data are represented as the means ± SD of three independent measurements

We simultaneously measured CO_2_ exchange, P700 absorbance, and chlorophyll fluorescence to calculate various photosynthetic parameters in the dark‐adapted leaves of wild‐type *A. thaliana* (Col‐0) and the mutants, *pgrl1* and *crr‐2*, under a sine‐like fluctuating light. This physiological measuring system was named Umibozu because the smoothly changing Y(ND) given by the sine curve‐like fluctuating light (described below) was like the shape of a traditional Japanese sea monster (‘Umibozu’, or sea goblin). During *slow* Umibozu, PFD of the light oscillated at a low frequency (6 per hour) like a sine curve in the range between a maximum (1,970 μmol photons m^−2^ s^−1^) and a minimum PFD (30 μmol photons m^−2^ s^−1^; Figure 1a). The frequency of fluctuating light in Umibozu is controlled by a software developed by Walz (Effeltrich, Germany) and can be flexibly modified from per second to per day; this enabled the evaluation of the tracking performance of P700 oxidation relative to the speed of light fluctuation. During *slow* Umibozu, photosynthetic parameters were determined every 1 min by the analysis with a saturated short‐pulse.

During *slow* Umibozu in Col‐0, *pgrl1*, and *crr‐2*, the uptake rate of CO_2_ was gradually enhanced with an increase in PFD (Figure 1b), indicating that rising PFD activates photosynthesis in Col‐0, *pgrl1*, and *crr‐2*. Moreover, photosynthesis was induced in response to light in a time‐dependent manner because we used dark‐adapted plant leaves in this study. That is, the increase in net CO_2_ assimilation rate involved the activation of photosynthesis dependent on both light intensity and illumination time, which is the reason why it was not proportional to fluctuating PFD particularly in the first 10 min (Figure 1b). To evaluate photosynthetic parameters before and after the photosynthesis induction, in this study we applied *slow* Umibozu to the plant leaves continuously three times for 30 min, i.e., 3 periods. Whereas in the first 10 min we could not observe any significant differences in the values among these plants, *pgrl1* finally showed the slightly lower CO_2_ assimilation rate than the other plants in the end of the measurement (Figure 1b).

Changing PFD in *slow* Umibozu caused the alterations of photosynthetic parameters in PSI in the *A. thaliana* wild‐type and mutants. Quantum yield of non‐photochemical energy dissipation due to donor side limitation of PSI, Y(ND), indicated the ratio of P700^+^ to total photo‐oxidizable P700 (i.e. P700 oxidation), as determined by spectrophotometric analysis (Harbinson & Hedley, 1993; Inoue et al., 1973; Schreiber & Klughammer, 2008). During *slow* Umibozu, the increase in PFD caused smooth increase in Y(ND) in Col‐0 and *crr‐2* (Figure 1c).We used the *A. thaliana* mutants, *pgrl1* and *crr‐2*, as the plants impaired in the electron acceptor side of PSI. As expected, we could not observe P700 oxidation in *pgrl1*; however, unexpectedly,*crr‐2* showed P700 oxidation such as Col‐0 in *slow* Umibozu (Figure 1c). The quantum yield of non‐photochemical energy dissipation due to acceptor side limitation, calculated as Y(NA), was remarkably higher in *pgrl1* than in Col‐0 and *crr‐2* (Figure 1d). The operating efficiency of PSI was calculated as the quantum yield of photochemical energy conversion in PSI, Y(I), which decreased with an increase in PFD in Col‐0, *pgrl1*, and *crr‐2* (Figure 1e). We found lower Y(I) in *pgrl1* than in Col‐0 and *crr‐2* in *slow* Umibozu (Figure 1e).

From chlorophyll fluorescence analysis, we calculated the photosynthetic parameters around PSII in *slow* Umibozu in Col‐0, *pgrl1*, and *crr‐2*. The oxidation of the acceptor side of PSII, inferred from photochemical quenching of chlorophyll fluorescence based on the lake model of the antenna system in PSII, qL, basically decreased and then increased inversely against PFD in all the three plants(Figure 1f). We note the possibility of free redox equilibration between PSII, PQ pool, and Cyt *b*_6_/*f*, which is the reason why qL is questioned as a proper indicator of PQ oxidation (Joliot, Lavergne, & Béal, 1992; Kirchhoff et al., 2000), might remain to be solved in future studies. The qL values were not different among these plants in the first 5 min; however, subsequently, it was lower in *pgrl1* than in Col‐0 and *crr‐2* (Figure 1f), which was presumably derived from the photo‐oxidative damage in PSI in *pgrl1* (Munekage et al., 2002; DalCorso et al., 2008; Suorsa et al., 2012; Kono & Terashima, 2016; Yamori et al., 2016; and Figure 4 in this study). In addition, we calculated NPQ during *slow* Umibozu. In Col‐0 and *crr‐2*, NPQ was rapidly induced by illumination with AL and then slowly increased until 7 min (Figure 1g). In *pgrl1*, NPQ required considerably longer time to be induced and was observed at lower levels in *slow* Umibozu than in Col‐0 and *crr‐2* (Figure 1g). The effective quantum yield of PSII, Y(II), was also calculated in *slow* Umibozu in these plants. We observed similar behaviors of Y(II) to those of qL in all the three plants (Figure 1h).

### 3.2 Responses of photosynthetic parameters to light fluctuation in *fast* Umibozu

**Figure 2:**
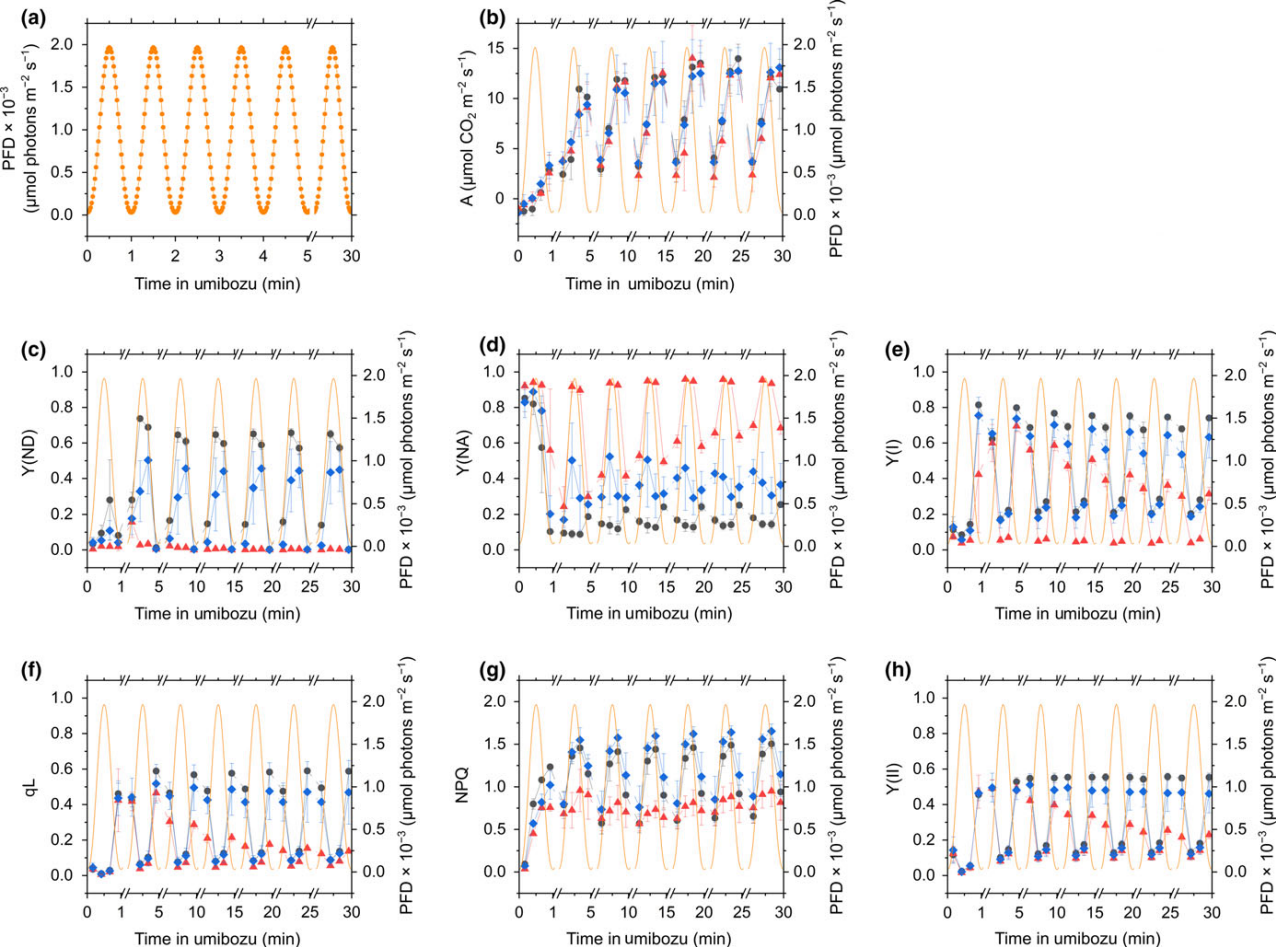
Time courses of photon flux density (PFD; a), net CO_2_ assimilation rate, A (b), Y(ND) (c), Y(NA) (d), Y(I) (e), qL (f), NPQ (g), Y(II) (h) during *fast* Umibozu in *Arabidopsis thaliana* wild‐type (Col‐0, black circles) and the mutants, *pgrl1* (red triangles) and *crr‐2* (blue diamonds). Orange lines indicate PFD. Data are represented as the means ± SD of three independent measurements

We applied *fast* Umibozu, where PFD of the light oscillated at a high frequency (60 per hour), to the dark‐adapted leaves of Col‐0, *pgrl1*, and *crr‐2*. In this experiment, PFD of AL changed in the range between the maximum (1,970 μmol photons m^−2^ s^−1^) and minimum values (30 μmol photons m^−2^ s^−1^) for 30 s (Figure 2a), resulting in 30 periods of a 1‐min sine‐like light fluctuation during the 30‐min measurement in *fast* Umibozu. To follow the rapidly fluctuating PFD during *fast* Umibozu, photosynthetic parameters were determined every 15 s in the first, 5th, 10th, 15th, 20th, 25th, and 30th periods of the 1‐min light fluctuation as shown in Figure 2.

As in *slow* Umibozu, we simultaneously measured gas exchange with P700 absorbance and chlorophyll fluorescence during *fast* Umibozu in Col‐0, *pgrl1*, and *crr‐2* and found little difference in the net CO_2_ assimilation rate among these plants (Figure 2b). We observed the lower CO_2_ assimilation rate for the first few minutes during *fast* Umibozu in these plants (Figure 2b), likely attributed to photosynthesis induction in the dark‐adapted plant leaves.

Unlike in *slow* Umibozu, in *fast* Umibozu, the induction of Y(ND) was hardly observed around the maximum PFD in the first period of the light fluctuation during *fast* Umibozu in Col‐0 and *crr‐2* (Figure 2c), indicating that the activation of electron sink, i.e., photosynthesis and photorespiration, in the transition from dark to light could not follow the rapid light fluctuation during *fast* Umibozu in this phase. Subsequently, Col‐0 could oxidize P700 in response to the rapidly fluctuating light (Figure 2c). However, *crr‐2* showed retarded induction of Y(ND) along with the increase in PFD, compared with that in Col‐0, throughout the 30‐min measurement in *fast* Umibozu (Figure 2c). In *pgrl1*, we could not detect Y(ND) (Figure 2c). The time courses of Y(ND) in these plants were alternatively shown as a raw trace of the relative amount of P700^+^ (Supporting Information Figure S1), clearly supporting the results in Figure 2c. In *pgrl1*, Y(NA) was remarkably higher throughout *fast* Umibozu, and *crr‐2* showed higher Y(NA) in the phases where PFD was rapidly rising, compared with that in Col‐0 (Figure 2d). Further, we found that Y(I) was lower in *pgrl1* than in Col‐0 and *crr‐2* (Figure 2e), as in *slow* Umibozu.

During *fast* Umibozu, qL inversely responded to the changes in PFD, and the values were largely and slightly lower respectively in *pgrl1* and *crr‐2* than in Col‐0 in the end of the 30‐min measurement (Figure 2f). We also measured NPQ during *fast* Umibozu and found that *pgrl1* could not induce NPQ in response to the rapidly fluctuating light, different from Col‐0 and *crr‐2* (Figure 2g). Furthermore, we found that Y(II) responded to *fast* Umibozu as well as qL in all the three plants (Figure 2h).

### 3.3 Effects of the frequency of fluctuating light on the relationships of qL and NPQ with P700 oxidation

**Figure 3:**
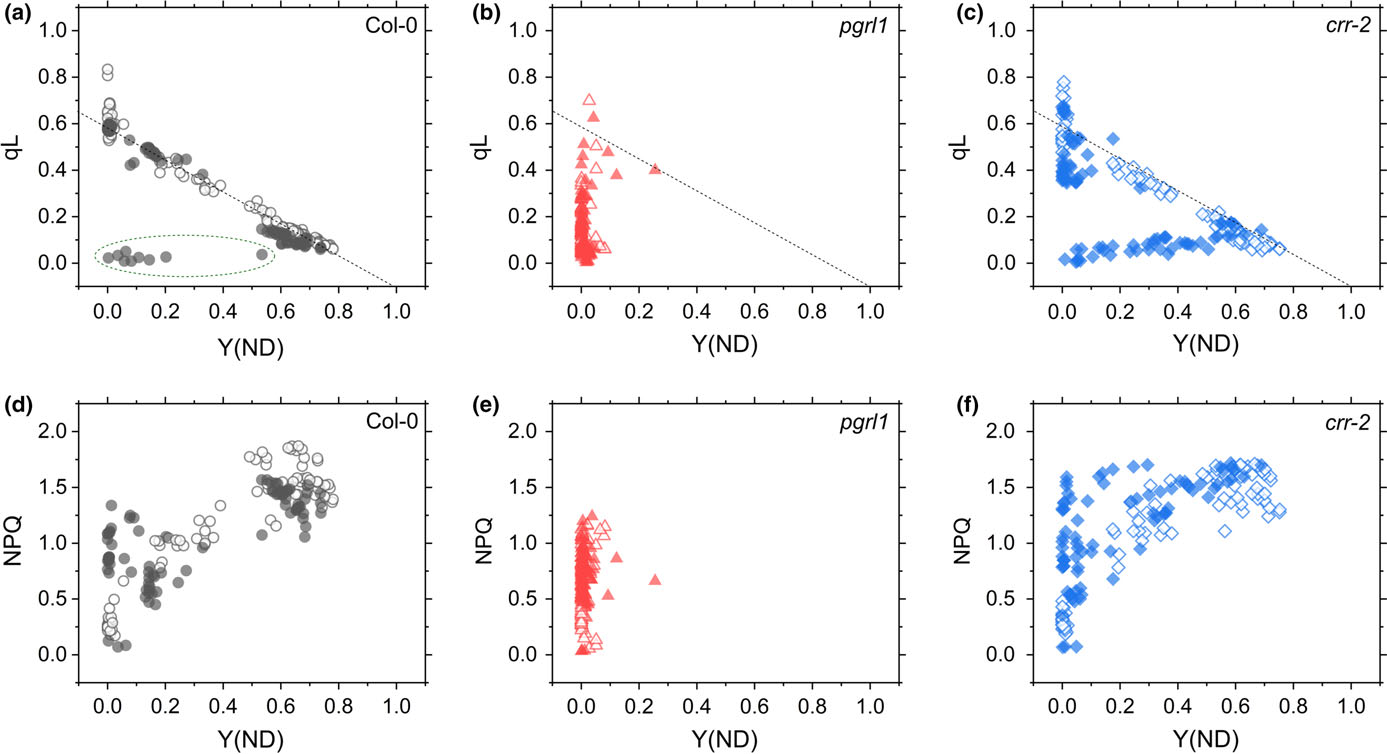
Relationships of qL (a‐c) and NPQ (d‐f) with Y(ND) during *slow* (open symbols) and *fast* (closed symbols) Umibozu in *Arabidopsis thaliana* wild‐type (Col‐0, a and d) and the mutants, *pgrl1* (b and e) and *crr‐2* (c and f). Dashed black line indicates the linear relationship between qL and Y(ND) during *slow* Umibozu in Col‐0. Dashed green frame surrounds the data in the first period during *fast* Umibozu in Col‐0. Data are derived from three independent measurements

We evaluated the relationships of qL and NPQ with Y(ND) during *slow* and *fast* Umibozu in Col‐0, *pgrl1*, and *crr‐2*. During *slow* Umibozu, we found a linear relationship between qL and Y(ND) in Col‐0 (indicated by dashed black line in Figure 3a‐c), implying that P700 oxidation was paralleled by the reduction of the electron acceptor side of PSII. The linear relationship was recognized in Col‐0 also during *fast* Umibozu except for the data in the first period of the light fluctuation (surrounded by dashed green frame in Figure 3a). Additionally, the relationship between qL and Y(ND) in *crr‐2* showed the similar linearity to that in Col‐0 during *slow* Umibozu, which was, nevertheless, broken during *fast* Umibozu even after the photosynthesis induction is nearly completed (Figure 3c). That is, *crr‐2* showed limited electron transport on the acceptor side of PSI in *fast* Umibozu, and the induction of P700 oxidation could not follow the rapid light fluctuation in *fast* Umibozu. In both *slow* and *fast* Umibozu, we failed to identify a clear relationship between NPQ and Y(ND) in Col‐0 and *crr‐2* although Y(ND) partially increased along with the induction of NPQ in some phases (Figure 3d,f). In *pgrl1*, we could not evaluate the relationships of these chlorophyll fluorescence parameters with P700 oxidation (Figure 3b,e), because we did not observe Y(ND) during both *slow* and *fast* Umibozu.

### 3.4 Photo‐oxidative damage in PSI during Umibozu

**Figure 4:**
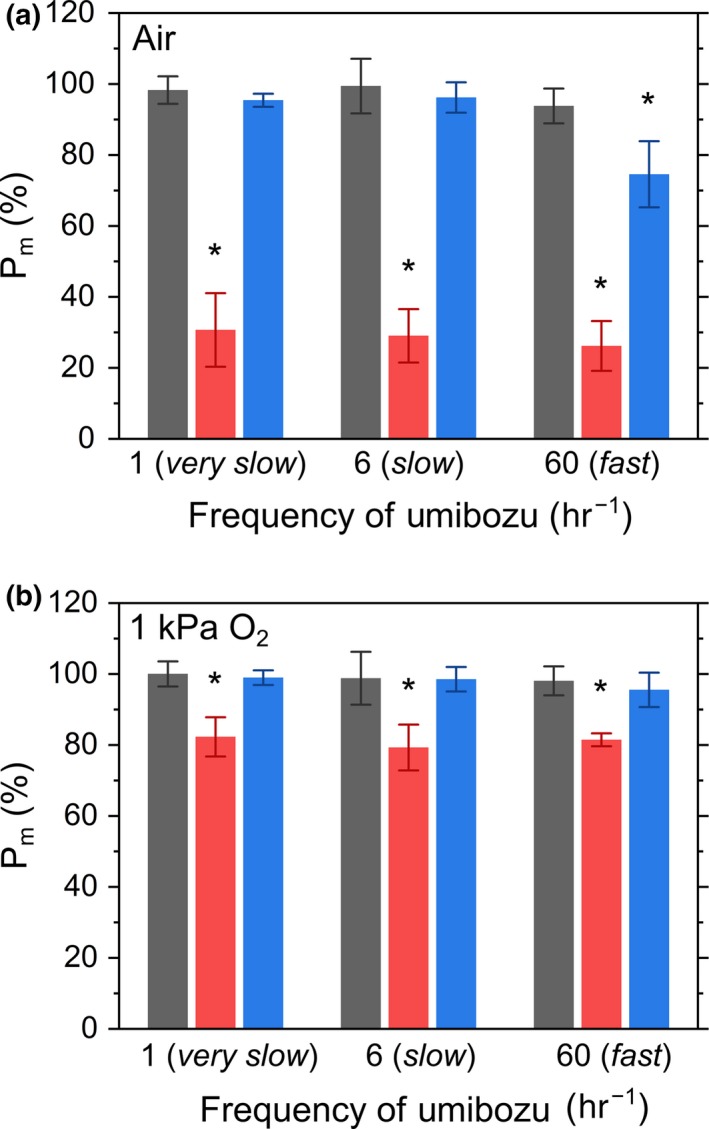
Decrease in *P*_m_ after Umibozu with different frequencies under ambient air (a) and 1 kPa O_2_ (b). *P*_m_ was obtained 30 min (in the dark) after 1 hr Umibozu in *Arabidopsis thaliana* wild‐type (Col‐0, gray) and the mutants, *pgrl1* (red) and *crr‐2* (blue). Data are represented as the means ± SD of three independent measurements. Asterisks indicate statistically significant differences (*p *<* *0.05) in the residual *P*_m_ of the mutants with Col‐0 as per Student's *t*‐test

Photon energy in the excess of the demand for photosynthesis causes the production of ROS, resulting in the inactivation of PSI in photosynthetic organisms unless P700 oxidation system operates. We exposed the dark‐adapted *A. thaliana* plant leaves of Col‐0, *pgrl1*, and *crr‐2* to Umibozu at three different frequencies (*very slow*, 1;*slow*, 6; and *fast*, 60 per hour) for 1 hr. In this experiment, the PFD of AL fluctuated from the minimum 30 to the maximum 1,970 μmol photons m^−2^ s^−1^ during the 1‐hr measurement. In addition, we did not apply any short saturation pulses, because repetitive short‐pulse illumination can cause PSI photoinhibition to mask the effect of the sine fluctuating light in Umibozu on PSI in plant leaves (Sejima et al., 2014). We evaluated the residual PSI activity of plant leaves as the ratio of total oxidizable P700 (*P*_m_) measured before and after Umibozu. In Col‐0, *P*_m_ did not decrease after Umibozu at all three frequencies (Figure 4a), which was in agreement with the experimental fact that Col‐0 can maintain P700 oxidized under both *slow* and *fast* Umibozu. Conversely, *pgrl1* showed a severe inactivation of PSI at all frequencies of Umibozu (Figure 4a). The mutant *crr‐2* showed inactivated PSI during *fast* Umibozu, but not during *slow* and *very slow* ones (Figure 4a). The decrease in *P*_m_ in *pgrl1* and *crr‐2* was alleviated by eliminating O_2_ to 1 kPa (Figure 4b). These data were consistent with the results of P700 oxidation in Umibozu (Figures 1 and 2) and support that P700 oxidation is required for the protection of PSI against the photo‐oxidative damage induced by ROS.

We also evaluated the residual PSII activity of plant leaves as the ratio of maximum quantum efficiency of PSII photochemistry (*F*_v_/*F*_m_) measured before and after Umibozu. To exclude the effects of both qE quenching and state transition on *F*_v_/*F*_m_, we incubated the plant leaves in the dark for 30 min after AL was turned off, and then *F*_v_/*F*_m_ values were evaluated. Compared with that in PSI, the decreases in PSII activity were small in these plants (Supporting Information Figure S2).

## 4 DISCUSSION

In the present study, we sought to investigate the impact of changing frequency of fluctuating light on a variety of photosynthetic parameters in Col‐0, *pgrl1*, and *crr‐2* during *slow* and *fast* Umibozu and verify the tracking performance of the molecular mechanisms for P700 oxidation in the C_3_ plant *A. thaliana*. Since the pioneering work of Eva‐Mari Aro's group (Suorsa et al., 2012), rectangular artificial fluctuating lights have been used in photosynthesis research on PSI photoinhibition (Allahverdiyeva et al., 2013; Gerotto et al., 2016; Kono & Terashima, 2016; Yamori et al., 2016). Compared with the rectangular fluctuating light, Umibozu provides a smoothly fluctuating artificial light. The smoothly fluctuating light has been utilized in preceding reports (Lucker, Hall, Zegarac, & Kramer, 2014). In this study, the most significant advantage of the sinusoidal change of PFD is to evaluate the tracking performance of photosynthetic parameters with a variety of light fluctuation by changing the frequency. In the present study, we adjusted the frequency of Umibozu to 6 (*slow*) and 60 per hour (*fast*). In Col‐0, P700 oxidation is rapidly induced with the support of donor side mechanisms of PSI during both *slow* and *fast* Umibozu as shown in the strong correlation between qL and Y(ND) except for the moments just after AL was turned on in *fast* Umibozu, i.e., an early phase of photosynthesis induction (Figure 3a). During *slow* Umibozu, we could observe small differences in the photosynthetic parameters between Col‐0 and *crr‐2* (Figure 1). Nevertheless, the electron acceptor side of PSI in *crr‐2* was remarkably limited on exposure to the rapidly rising PFD in *fast* Umibozu as reflected in Y(NA) (Figure 2d), resulting in the retardation of the induction of P700 oxidation (Figure 2c). Finally, the lack of NDH caused PSI photoinhibition only in *fast* Umibozu, but not in *slow* and *very slow* ones (Figure 4). That is, the tracking performance of *A. thaliana* to induce P700 oxidation against rapidly fluctuating light is supported by a NDH‐dependent mechanism. Recently, C_3_ plant mutants deficient in NDH have been reported to be impaired in P700 oxidation and to show PSI photoinhibition under a rectangular artificial fluctuating light (Kono & Terashima, 2016; Yamori et al., 2016). The rectangular light fluctuation exposes plant leaves to instantaneous changes in PFD of AL, which can be defined as a super‐*fast* fluctuating light. Therefore, the inability to oxidize P700 in *crr* mutants under the rectangular fluctuating light also supports the conclusion of the present study that NDH is required for the rapid start of P700 oxidation in C_3_ plant leaves. On the other hand, in *pgrl1* P700 oxidation was not observed during both *slow* and *fast* Umibozu (Figures 1 and 2), and the photo‐oxidative damage in PSI was not affected by the frequency of the fluctuating light in Umibozu (Figure 4). These data suggest that a PGR5/PGRL1‐dependent mechanism plays a critical role to keep P700 oxidized regardless of the frequency of fluctuating light. Overall, changing frequency of fluctuating light in Umibozu provides a novel insight into the mechanism of P700 oxidation in plants under natural environmental variations with different durations and frequencies of light (Pfitsch & Pearcy, 1989).

Here we rethink the reason why PGR5/PGRL1 and NDH are required for P700 oxidation in *A. thaliana*. The most popular hypothesis regarding the inability to oxidize P700 in *pgrl1* and *crr‐2* is likely to be based on the CET model, in which electrons are donated from the acceptor side of PSI back to PQ through the PGR5/PGRL1 and NDH pathways, respectively, to promote lumen acidification (Kono & Terashima, 2016; Nandha et al., 2007; Yamori et al., 2016). Recently, Mosebach et al. (2017) showed that, in the green alga *Chlamydomonas reinhardtii*, the lack of PGR5 and PGRL1 impairs the association of Fd‐NADP^+^ reductase with the thylakoid membrane to lower the reduction rate of NADP^+^ to NADPH in vitro, which also supports the results of *pgrl1* in this study. Further, in the case of NDH we note another possibility that NDH oxidize Fd in the dark or weak light for the rapid start of P700 oxidation in response to rapidly rising PFD in the process of chlororespiration (Feilke et al., 2016). Under light conditions, Fd is oxidized by Fd‐NADP^+^ reductase to produce NADPH for driving the Calvin–Benson cycle. However, the change of the turnover of the Calvin‐Benson cycle requires a longer time, compared with the alteration of photosynthetic electron transport reaction, which might require the oxidation of Fd by chlororespiration for the alleviation of the electron acceptor side limitation in PSI in *fast* Umibozu (Figure 2c). Unlike in *fast* Umibozu, the alteration of the electron sink capacity in the Calvin‐Benson cycle might be in time for the induction of P700 oxidation in *slow* Umibozu (Figure 1c).

The linear relationship between qL and Y(ND), which were calculated using completely different experimental approaches (Figure 3a) strongly supports that photosynthetic linear electron flow is basically limited at the step between PSII and PSI, i.e., Cyt *b*_6_/*f*. Cyt *b*_6_/*f* is known to be the limiting step of photosynthetic linear electron flow without any specific regulatory mechanism, because the oxidation of PQH_2_ is the slowest step in the photosynthetic electron transport system, and the amount of Cyt *b*_6_/*f* is normally smaller than those of PSII and PSI in plant leaves (Anderson, 1992; Kirchhoff et al., 2000; Schöttler & Tóth, 2014; Schöttler et al., 2015; Stiehl & Witt, 1969; Yamori, Evans, & von Caemmerer, 2010; Yamori et al., 2011). However, under fluctuating environments, plants require regulatory mechanism (i.e. P700 oxidation system) to oxidize P700 for the protection of PSI against ROS damage (Shimakawa, Shaku et al., 2016; Shimakawa, Akimoto et al., 2016; Shimakawa, Ishizaki et al., 2017; Takagi et al., 2017), which is clearly evidenced by the flexible response of Y(ND) to environmental variations. At present, ΔpH across the thylakoid membrane is believed to regulate the oxidation activity of PQH_2_ in Cyt *b*_6_/*f* for the induction of P700 oxidation in response to fluctuating environments. According to existing theories (Kanazawa & Kramer, 2002), photon energy drives photosynthetic linear electron transport to form ΔpH, which then down‐regulates photosynthetic electron transport from PSII to PSI by stimulating the suppression of electron transport in Cyt *b*_6_/*f* and inducing qE quenching (termed as photosynthetic control; Schöttler et al., 2015 and references therein). In addition, the accumulation of H^+^ on the luminal side of the thylakoid membrane is also supported by the regulation of CET (Nandha et al., 2007) and g_H_^+^ (Armbruster et al., 2014; Rott et al., 2011; Takizawa et al., 2008). In C_3_ plant leaves, NPQ originates qE quenching, state transition, and PSII photoinhibition, which have different relaxation times (Ruban, 2016). We observed a lag of few minutes between the decreases in PFD and NPQ during *slow* Umibozu in Col‐0 (Figure 2e), which is consistent with the common explanation that NPQ in C_3_ plants is mainly caused by qE quenching dependent on ΔpH across the thylakoid membrane (Kanazawa & Kramer, 2002; Ruban, 2016). However, we could not identify the proportional linear relationship of NPQ with Y(ND) during both *slow* and *fast* Umibozu in Col‐0 (Figure 3d), indicating that the relationships among NPQ, Y(ND), and ΔpH are complicated in the intact plant leaves (Hald, Nandha, Gallois, & Johnson, 2008). Alternatively, a novel regulatory theory for P700 oxidation, RISE (Shaku et al., 2016; Shimakawa et al., 2018), may provide some insights into the understandings of these facts.

Notably, Y(I) in *pgrl1* was lower than those in Col‐0 and *crr‐2* where Y(II) in *pgrl1* was like those during both *slow* and *fast* Umibozu (Figures 1 and 2). First, we can consider that the Y(I) uncoupled with Y(II) is derived from CET dependent on PGR5/PGRL1. As a second possibility, Y(I) does not properly reflect the efficiency of the electron transport at PSI. Sacksteder and Kramer (2000) suggested that the total electron transport activity in PSI is determined by the summation of the estimations of electron fluxes through cytochrome*f*, plastocyanin, and P700. Finally, the electron transport reaction from the electron acceptor side of PSI back to P700^+^ possesses μs‐ or ms‐ order half times (i.e. the charge recombination of P700^+^ with F_X_ exhibits 0.5–1.5 ms of the half time; Semenov et al., 2000), which can cause an overestimation of Y(I), particularly when P700^+^ is accumulated. The charge recombination of P700^+^ results in the dissipation of excess electrons on the electron acceptor side of PSI, which might be one of how P700 oxidation protects PSI against ROS damage.

## CONFLICT OF INTEREST

The authors have no conflict of interest to declare.

## AUTHOR CONTRIBUTIONS

C.M. conceived the original screening and research plans; C.M. supervised the experiments; G.S. performed all the experiments; C.M. and G.S. designed the experiments and analyzed the data; C.M. and G.S. conceived the project and drafted the manuscript.
