# Supplementary material for: Changing frequency of fluctuating light reveals the molecular mechanism for P700 oxidation in plant leaves
Source: Plant Direct. 2018 Jul 23;2(7):e00073. doi: 10.1002/pld3.73 (PMC6508772; doi:10.1002/pld3.73)
Supplement: Supplementary file 1 [file PLD3-2-e00073-s001.pdf]

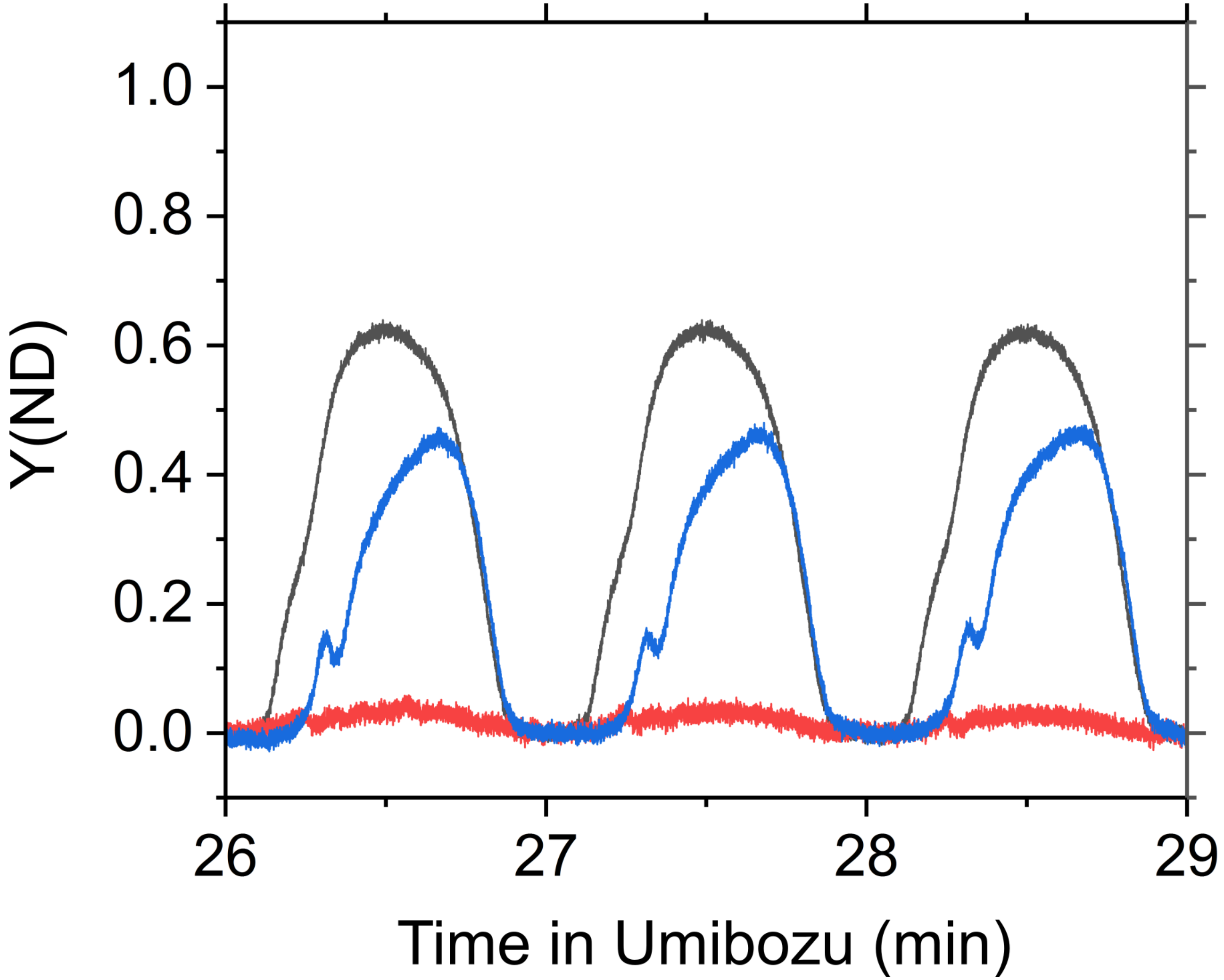

Fig. S1. Time courses of the relative amount of P700^{+} during *fast* Umibozu in *Arabidopsis thaliana* wild-type (Col-0, grey) and the mutants, *pgrl1* (red) and *crr-2* (blue). The signals are normalized at the total oxidizable P700 (i.e., P_m) as 1.0. Data are shown as the representative data of three independent measurements.

Figure S1. Shimakawa et al.
