# Supplementary material for: Changing frequency of fluctuating light reveals the molecular mechanism for P700 oxidation in plant leaves
Source: Plant Direct. 2018 Jul 23;2(7):e00073. doi: 10.1002/pld3.73 (PMC6508772; doi:10.1002/pld3.73)
Supplement: Supplementary file 2 [file PLD3-2-e00073-s002.pdf]

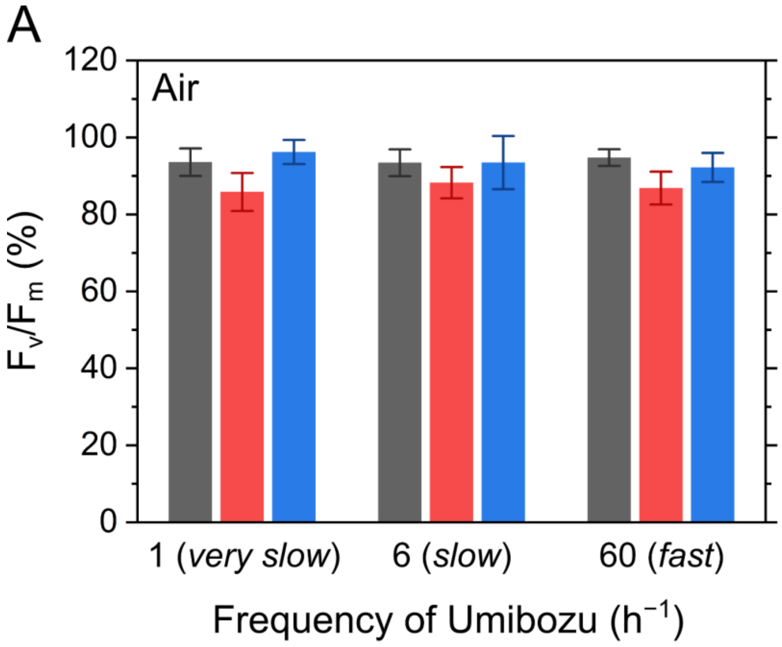

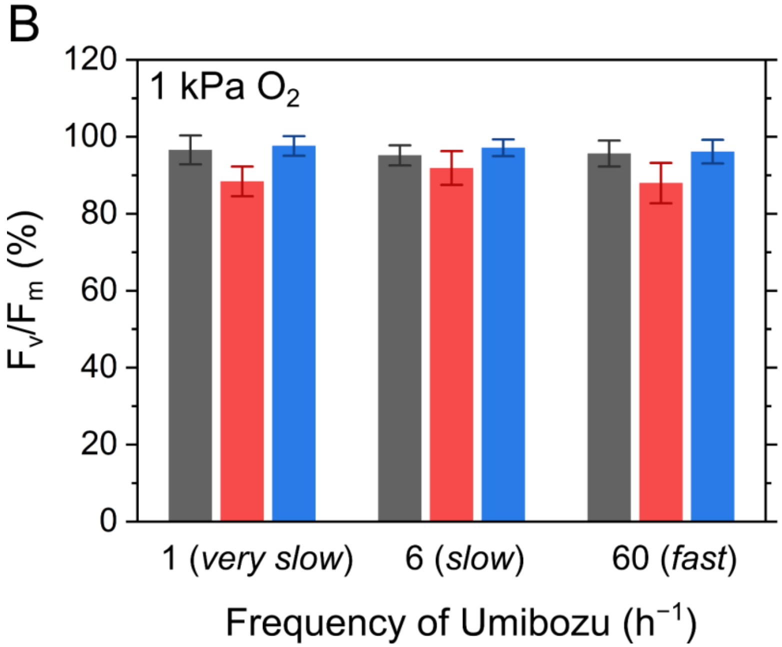

Fig. S2. Decrease in F_v/F_m after Umibozu with different frequencies under ambient air (A) and 1 kPa O_2 (B). F_v/F_m was obtained 30 min (in the dark) after 1 h Umibozu in *Arabidopsis thaliana* wild-type (Col-0, grey), and the mutants, *pgrl1* (red) and *crr-2* (blue). Data are represented as the means ± standard deviations of three independent measurements.

Figure S2. Shimakawa et al.
