# Supplementary material for: Changing frequency of fluctuating light reveals the molecular mechanism for P700 oxidation in plant leaves
Source: Plant Direct. 2018 Jul 23;2(7):e00073. doi: 10.1002/pld3.73 (PMC6508772; doi:10.1002/pld3.73)
Supplement: Supplementary file 3 [file PLD3-2-e00073-s003.pdf]

Response to Reviewer 1

We thank you for reviewing our manuscript and providing helpful suggestions. We have revised our manuscript following your advice.

----

1) Most importantly, I have severe concerns about the experimental design chosen by the authors, which seems to be unnecessarily complicated by overlaying two different processes: A sinus-oscillation in actinic light intensity, and photosynthetic induction of dark-adapted leaves. Because the authors start the experiment with dark-adapted plants, and perform their measurements only for 10 min in the light, during the entire time of the experiment, effects due to the oscillating light intensity and due to the different phases of the photosynthetic induction process add up to the observed effects. The authors argue that photosynthetic induction only plays a role during the first cycle of the slow Umibozu experiment, but this is likely not correct. For example, they should take a close look at the work done by the Stitt group in the 1990s to elucidate the different phases of Calvin cycle activation and its balancing to the subsequent reactions of dark metabolism such as starch and sucrose biosynthesis.

Instead, to avoid these unnecessary complications, the authors should have determined the relevant photosynthetic parameters such as F0 and FM in dark-adapted leaves, then should have pre-illuminated the leaves at an intermediate light intensity for 15 to 30 min, to allow a full activation of the Calvin cycle, before starting their light oscillation regime. Also, I do not understand the rational for lowering the actinic light intensity to zero during the treatment, as this in non-physiological. On a cloudy day, light may decrease rapidly by more than an order of magnitude, but never decreases to zero. Why do the authors not maintain a minimum light intensity instead of decreasing the light

intensity to zero?

>>> In this revision, we re-established the experimental design, and performed all experiments again. First, we applied Umibozu for 30 min to the plant leaves, which enables us to evaluate the photosynthetic parameters during *slow* and *fast* Umibozu in Col-0, *pgrl1*, and *crr-2* both before and after the activation of the Calvin-Benson cycle. Second, the photon flux density of the actinic light during Umibozu was adjusted to the range from the minimum 30 to the maximum 1970 μmol photons m^{-2} s^{-1} in reference to your advice.

----

2) Concerning the general novelty of the experimental design, the authors claim multiple times that their "Umibozu" experiments are novel. For example, they dedicate Figure 1A to this postulate, writing that this is "a novel concept of physiological measurements". In a similar way, on page 29 in the discussion, they state that they "established a novel concept of physiological measurements with Umibozu". This is wrong. The authors use the commercial Dual-PAM systems from Walz for their measurements, which contain the sinus modulation function used in these experiments since years as a standard feature. Not only has this feature been used by several groups before, but also other groups such as the Kramer group have used similar schemes of sinus modulations of actinic light intensity a lot in the last few years. The authors fail to cite any of these articles. Using an existing, established commercial technique not developed by them to postulate novelty of the experimental approach and even coin a novel name for it is astonishing. There are also several other such examples of claims of priority and novelty, which are not justified. The authors should read the existing literature more carefully.

>>> We totally revised the manuscript, and removed the multiple claims for the novelty of using

sinus fluctuating light. Additionally, we cited Lucker et al. (2014) *Algal Research* 6, 242-249 in the Discussion section in the revised manuscript (line 301−302). Further, we removed Fig. 1.

----

3) There are also multiple statements on results in the text, which are not sufficiently or not at all supported by experimental data. On page 11, line 232, the authors write: "ECSt depends on properties of the leaves, and not only the density of chloroplasts, but also the content of light-harvesting complexes that harbor the shifted experiments". This is well known for decades, but the authors neither cite any suitable literature here (work by, for example: Horst Witt, Wolfgang Junge, Peter Jahns, Antony Crofts), nor do they present any of their own data to address the question whether or not LHCII and pigment contents are altered in any of their mutants. This would be a minimum requisite to compare ECSt measurements between the wild type Col-0 and the two mutants. Anyhow, because all relevant regulatory mechanisms in the thylakoid lumen such as "photosynthetic control" or NPQ are only dependent on the delta pH component of the proton motive force, ECSt data are not informative in this manuscript. Only a correct determination of pmf partitioning would help with the interpretation of these data.

>>> In reference to both your comment and the Reviewer 2's decision, we have concluded that the ECSt data have little impact on our manuscript. As you suggest, a correct partitioning of ECSt probably help us to interpret our data. However, it needs a switching actinic light off over 30 s (Cruz et al., 2001 *Biochemistry* 40, 1226-1237), which is not preferable during the Umibozu measurement. From these facts, we would like to remove ECSt data in the revised manuscript, and focus on the effects of changing frequency of fluctuating light on photosynthetic parameters.

----

4) I do not understand the ferredoxin data provided by the authors. The authors give ratios for total ferredoxin to total P700 on page 11, line 221 to 223. They give values of 0.17 for the wild type and both mutants. This is dramatically too low. Normally, ratios between one to three ferredoxins per P700 are given in the literature. How did the authors obtain these results? They just state that they calculated ratios from transmittance, but never provide the difference extinction coefficients needed to calculate the correct ratios from the transmittance changes. The authors need to check these data (do they give P700 / ferredoxin ratios - that would come closer to the expected range).

>>> The redox states of ferredoxin and P700 was spectroscopically measured with Dual-KLAS-NIR (Klughammer and Schreiber, 2016 *Photosynthesis Research* 128, 195-214). Both transmittances were relative values, which is the reason why the ratio of these transmittances are not reasonable in a comparison with the predicted absolute values. Therefore, we cannot quantitatively evaluate the amounts of reduced ferredoxin and oxidized P700, but measure only the ratios of the reduced (or oxidized) forms to the total amounts. This method has been already published (Klughammer and Schreiber, 2016 *Photosynthesis Research* 128, 195-214), but certainly might not have been yet well established as your advice and the Reviewer 2's comment. We decided to omit the ferredoxin data in the revised manuscript, because they are not essential for the significance of the present study.

----

5) Finally, I do not believe that the RISE mechanism exists. The data suggested to support such a scenario have very likely been misinterpreted, and key experiments are missing. The kinetics of the different redox reactions within the cytochrome b6f complex have been studied for decades, and it is well established that the linear branch is rate-limiting, especially plastoquinol re-oxidation, but also the movement of the Rieske protein from the

quinol binding site towards cytochrome f. This linear section of electron transport in the cytochrome b6f complex is also subject to the "photosynthetic control" mechanism. The Q-cycle has never been implicated to be rate-limiting. Based on the existing kinetic models of the cytochrome b6f complex, it is exceptionally unlikely that both b-type hemes and heme ci could get reduced, which would be expected in case of the RISE model. This has only been observed in the presence of specific inhibitors such as NQNO. The main result postulated by the authors to support the RISE mechanism is the correlation between qP, which the authors use as a measure for the redox state of the plastoquinone pool, and the donor-side limitation of PSI. However, such a correlation is an expected consequence of the rate-limiting function of plastoquinol re-oxidation: Electrons accumulate in the plastoquinone pool and on the PSII acceptor side, and the high potential chain including P700 becomes oxidized. So the observed correlation is a trivial downstream consequence of the normal rate-limiting function of the cytochrome b6f complex, and no indication whatsoever for a limitation in the Q cycle. Exactly the same relationship between qP and Y(ND) reported here under fluctuating light can be seen in standard slow light response curve of photosynthetic electron transport. If the authors want to ultimately proof (or more likely disproof) their RISE model, they should measure the redox states of the b-type hemes by difference absorbance spectroscopy and / or the redox state of the heme ci by EPR. To confirm the RISE mechanism, the authors need to detect a quantitative reduction of all three hemes. As long as that is not the case (and is has never been observed, despite of decades of investigation into the cytochrome b6f complex), the RISE concept would be valid.

>>> We totally revised the manuscript, including the Title, Abstract, Introduction, Results, and Discussion sections. We removed many statements about RISE, and only noted the possibility

of the existence (line 377−378).

---

6) A minor point is that the authors have to be much more careful about the interpretation of qP: This is no direct measure for the redox state of the plastoquinone pool, it only is an approximation for the redox state of the PSII acceptor side, and likely, less accurate than the newer qL parameter, which the authors should use instead. Because both qP and qL are only measures of the redox state of QA, several assumptions are needed to calculate the redox state of the PQ pool. The authors mention a few of these problems (line 443), but still should be much more careful in the way they equate qP to the redox state of the plastoquinone pool.

>>> We used qL but not qP as the parameter for the electron acceptor side of PSII in the revised manuscript. Additionally, we revised some explanations (line 193−199, 250−252) to suggest that qL (or qP) is an indicator for the oxidation of Q_A but not the plastoquinone pool.

Response to Reviewer 2

We thank you for reviewing our manuscript and providing many helpful suggestions. We have revised our manuscript following your advice.

In this paper the authors put forward an approach to studying plant photosynthetic responses, which they call Umibozu, which involves exposed leaves to fluctuating light following a sine wave pattern. This has the potential to bring out information relating to the dynamic responses of plants to changes in light. The authors highlight 2 main findings. Firstly, they claim a correlation between what they call plastoquinone redox state (in fact qP, a measure of QA redox state, according to one of 2 possible models for PSII) and P700 oxidation. This correlation is however only valid under certain conditions, during the relaxation of signals with slow changes. The relationship is not robust and there is no evidence presented that there is a causal relationship. Secondly, they found a non-correlation between non photochemical quenching and a signal called the electrochromic shift. This latter observation is not surprising, since there is no simple link between these phenomena, though both are in part determined by the membrane pH gradient.

>>> We totally revised the manuscript to focus on the results of the effects of changing frequency of fluctuating light on photosynthetic parameters in the C_3 plant *Arabidopsis thaliana* and the mutants *pgrl1* and *crr-2*. The relationship of P700 oxidation with qL is still suggested in the revised manuscript, but we rewrote in reference to your advices (line 349−378).

The work is carefully designed and the results are mostly clearly presented (though some complex figures are hard to read). Some of the figure legends are incomplete. There are

some important technical questions, notably the impact of rapidly repeated flashes, which the authors do address. I am concerned that for some of the data, the authors are too ready to accept what it says in the manual of the machines they have bought. The ECS signal is complex and the deconvolution of it is non trivial and must be approached critically. The ferredoxin signal is also not a well established method and I have some concerns about its rather naive application.

>>> First, we revised Figures and the legends more clearly. Second, we re-established the experimental design to perform all experiments in this revision. In this process, we limited the use of a saturated short-pulse smaller during Umibozu. Finally, in reference to both your comment and the Reviewer 1's decision we would like to remove ECSt and Fd data in the revised manuscript.

The paper is generally well written with few errors of English. The data are complex and the description often difficult to follow, even for a relatively expert reviewer. I felt that the discussion was much too long and too speculative. I think much more focus is needed on the conclusions which can be drawn from the data presented is required, and an appraisal of how the methodology adds to our understanding, rather than more general discussion of the regulation of electron transport. In concluding, they need to make a stronger case that their method adds information which has not already been gained from more conventional approached.

>>> We revised the manuscript to shorten the Discussion section and emphasize the conclusion of this study, i.e., the effects of changing frequency of fluctuating light on photosynthetic parameters in the photosynthetic physiological measurements in *A. thaliana* plant leaves.

Lines 82-123: This discussion should consider including reference to the work of Hald et al., 2008, BBA, 1777, 433-440, who showed evidence for non-correlation between pH gradient and P700 reduction kinetics and presented evidence that cyt bf turnover is regulated in response to NADP/H redox state.

>>> We revised the paragraph with the indicated citation (line 374−377).

Lines 124-158: I find this paragraph is too long and detailed. Fig 1 and the explanation of the method would be better places in the methods or results section. The introduction can close with a brief overview of the experimental approach and conclusions but at a much less detailed level. The authors could however consider moving the explanation for the name they use from lines 176-178 to here. Also, I did not understand Fig1A and I am not sure that it adds anything to the paper.

>>> We moved the explanations of our experimental design from the Introduction to the Results sections in reference to your advices (line 153−165). Further, we removed Fig. 1.

Figure 2: qP is not a measure of the PQ pool redox state and should not be explained as such. It is one possible measure of the redox state of QA. This is an especially important distinction in the case of this experimental design as the system is non-equilibrium. Also, why have the authors expressed data as qP, rather than qL,(as per Kramer et al., 2004) which is probably a better representation of the situation in Arabidopsis?

>>> We used qL but not qP as the parameter for the electron acceptor side of PSII in the revised

manuscript. Additionally, we revised some explanations (line 193−199, 250−252) to suggest that qL (or qP) is an indicator for the oxidation of Q_A but not the plastoquinone pool.

I am concerned about the measurements of ECS which do not seem to have a firm theoretical basis. This is not a signal which has a single simple interpretation and I think it requires a more nuanced discussion. The signal seen in a 1 sec decay is complex, including both ΔpH and potential difference, as the authors discuss. The measurements shown do not include any attempt to deconvolute these different components. It may be possible to consider the decay kinetics for the signal, which Kramer has claimed as an indicator of flux through the ATPase (gH+), though other interpretations are available. In view of these points, I am not convinced that including these data add greatly to the data.

>>> In reference to both your comment and the Reviewer 1's decision, we have concluded that the ECSt data have little impact on our manuscript. Partitioning of ECSt may improve our interpretations, but it needs a switching actinic light off for approximately 30 s (Cruz et al., 2001 *Biochemistry* 40, 1226-1237), which is very difficult for the present Umibozu measurement. From these facts, we would like to remove ECSt data in the revised manuscript, and focus on the effects of changing frequency of fluctuating light on photosynthetic parameters as shown in the title of the manuscript.

Figure S1 - the legend does not match the figure. It is not made clear which colour line represents which signal. Legend only references the Fd signal, not the P700. The calibration of the Fd signal is unclear to me. It is suggested that 500 µmol.m-2.s-1 of light was applied to a dark adapted leaf. The authors offer no evidence that this is induces of 100% of Fd reduction. Optical measurements of Fd redox state in optically complex

samples such as leaves should be approached with great caution and appropriate controls are essential to ensure that you are measuring what you think you are measuring.

>>> The maximum reduction level of ferredoxin was spectroscopically determined by the illumination with an actinic light (500 μmol photons m^{-2} s^{-1}) in the dark-adapted plant leaves, which is a default protocol in Dual-KLAS-NIR (Walz, Effeltrich, Germany) proposed by the publication (Klughammer and Schreiber, 2016 *Photosynthesis Research* 128, 195-214). However, this method might not have been yet well established as you suggested. In reference to your advice and the Reviewer 1's suggestion, we decided to omit the ferredoxin data in the revised manuscript, because they are not essential for the significance of the present study.

Figure S2 D-F it is not indicated in the legend what the different symbols represent

----

>>> We removed the indicated figure, because we applied the Umibozu measurement to the plant leaves for 30 min in the revised manuscript.

Figure 3 - it is rather difficult in some cases to work out the sequence of points. A stronger colour gradient or e.g. following a colour change, rather than simply a shade change may make this easier. Or possibly annotating with arrows?

>>> In the revised manuscript, we applied the Umibozu measurements to the plant leaves for 30 min, i.e., 3 or 30 periods. Therefore, the color gradient may rather make the figures more complicated. In this revision, we used the same color for all data in the same measurement each other (wild-type, black; *pgrl1*, red; *crr-2*, blue).

Line 290: "although the values were greater in rETR(I) than in rETR(II)" It is meaningless to compare relative ETR rates, as the units on these are arbitrary and different.

>>> In the revised manuscript, we omitted the evaluation of rETR in reference to your advice.

Line 293-4: "These data supported that P700 oxidation was strongly correlated with the reduction of the PQ pool" You have not measured PQ redox state. The correlation shown in Fig 3A anyway is not a simple relationship - there is a reasonable correlation between qP and Y(ND) over part of the curve (decreasing light, except at the lowest irradiances, but not during the induction phase. This implies that there is no causal relationship between these parameters.

>>> First, we revised some explanations (line 193−199, 250−252) to suggest that qL (or qP) is an indicator for the oxidation of Q_A but not the plastoquinone pool.

Second, we would like to note that the correlation between Y(ND) and qL is conserved as long as the electron acceptor side of PSI is not limited (line 225−229, 305−309). In the early phase of photosynthesis induction such as just after AL was turned on, the activation of electron sink, i.e., photosynthesis and photorespiration, in the transition from dark to light was assumed not to follow the rapid light fluctuation during *fast* Umibozu.

Figure S4 - The calculation used in this figure is not valid. The relative ETRs in the two cases are in different units. Also, it is not clear to me how the authors have estimated ETR1 in the pgrl1, which is acceptor side not donor side limited.

>>> In the revised manuscript, we omitted the evaluation of rETR in reference to your advice.
